# Measuring Potential Dermal Transfer of a Pesticide to Children in a Child Care Center

**DOI:** 10.1289/ehp.8283

**Published:** 2005-09-20

**Authors:** Elaine A. Cohen Hubal, Peter P. Egeghy, Kelly W. Leovic, Gerry G. Akland

**Affiliations:** 1 National Center for Computational Toxicology, and; 2 National Exposure Research Laboratory, U.S. Environmental Protection Agency, Research Triangle Park, North Carolina, USA; 3 Private Consultant, Raleigh, North Carolina, USA

**Keywords:** children, dermal exposure assessment, dermal-transfer coefficients, FQPA, pesticide exposure

## Abstract

Currently, the major determinants of children’s exposure to pesticides are not fully understood, and approaches for measuring and assessing dermal exposure in a residential setting have not been sufficiently evaluated. In one approach, dermal exposure is estimated using empirically derived transfer coefficients. To assess the feasibility of using this approach for assessing children’s exposure to pesticides, we conducted a study was conducted in a child care center that had a preexisting contract with a pest control service for regular monthly pesticide applications. Children in the selected child care center were monitored using full-body cotton garments to measure dermal loading. Pesticide residues on classroom surfaces were measured in the areas where the children spent time. Measured surface-wipe loadings ranged from 0.47 to 120 ng/cm^2^, and total garment loadings ranged from 0.5 to 660 pg/cm^2^. The garment and surface loading measurements were used to calculate dermal-transfer coefficients for use in assessing children’s residential exposure to pesticides. Dermal-transfer coefficients calculated using these data range from approximately 10 to 6,000 cm^2^/hr. The wide range in these values demonstrates the importance of developing standard surface-measurement protocols if this approach is to be used to assess dermal exposure in a residential environment. The upper-range values resulting from this study were found to be similar to the default value used by the U.S. Environmental Protection Agency to assess children’s dermal exposures resulting from contact with indoor surfaces.

The Food Quality Protection Act of 1996 requires that children’s risks to pesticide exposures from all sources be considered during the tolerance-setting process. An initial assessment of critical exposure pathways for children indicates that dermal contact may result in high residential exposures to pesticides (Cohen Hubal et al. 2000b). Currently, because of insufficient data on children’s exposures and activities, quantitative assessments must rely heavily on default assumptions as substitutes for missing information (Cohen Hubal et al. 2000a, 2000b). In addition, the major determinants of children’s exposure are not fully understood, and approaches for measuring and assessing dermal exposure in a residential setting have not been sufficiently evaluated.

Currently, the U.S. Environmental Protection Agency (EPA) Office of Pesticide Programs (OPP) uses a transfer-coefficient approach to assess children’s residential exposures to pesticides (U.S. EPA 1997, 1999, 2001a). In this type of assessment approach, exposure is estimated using empirically derived dermal-transfer coefficients to aggregate the mass transfer associated with a series of contacts with a contaminated medium. The transfer-coefficient approach was developed to assess occupational exposure in an agricultural setting where workers are engaged in similar activities and are exposed to relatively homogeneous environmental concentrations of pesticides. With this approach, dermal exposure sampling using a surrogate-skin technique such as a patch sampler or a whole-body garment sampler is conducted simultaneously with surface sampling for a specific activity (e.g., harvesting apples). A dermal-transfer coefficient is then calculated for this work activity. In later studies, this transfer coefficient can then be used to estimate exposure for a similar activity by collecting only surface samples (Fenske 1993).

Although pesticide levels in a residential environment are likely to be nonuniform and resident activities varied, this approach has also been used to assess residential exposure to pesticides. Dermal-transfer coefficients for assessing residential exposures to pesticides have been developed previously (Formoli 1996; Ross et al. 1990, 1991; Vacarro et al. 1996). In these studies, transfer coefficients are developed for adults performing choreographed reproducible activities upon reentry after a pesticide application. Currently, the U.S. EPA OPP uses a set of default dermal-transfer coefficients to conduct residential exposure assessments. For indoor surfaces, the default value for adults is 16,700 cm^2^/hr, and the value for children 1–6 years of age is 6,000 cm^2^/hr. These values are based on the previously reported studies conducted with adults; to develop values for children, the adult values were scaled by the average body surface area for 1- to 6-year-olds (U.S. EPA 1997, 1999, 2001a).

To date, only limited research has been conducted to develop transfer coefficients for children (Black and Fenske 1996). Black and Fenske (1996) evaluated transfer of a fluorescent tracer to children 4–9 years of age engaged in outdoor activities on turf. Transfer coefficients ranging from 3,000 to 16,000 cm^2^/hr were reported. However, transfer coefficients have not been developed specifically for very young children in an indoor environment, and the feasibility of applying this approach to assess children’s dermal exposures in a residential environment has not been evaluated sufficiently.

Because of ethical concerns, there is a very reasonable reluctance in the research community to allow participation of children in postapplication exposure-monitoring studies. However, in many parts of the country residential and institutional consumers contract with professional pest control operators to apply pesticides in the home, office, or school at regularly scheduled intervals. This type of service is particularly popular in the southeastern United States, where the mild winter conditions produce significant pest control problems. Therefore, many children live and go to school in facilities that are treated with pesticides on a regular schedule determined by the pest control operator under contract with the consumer. To develop dermal-transfer coefficients for young children, we conducted this study in a child care center that had a pre-existing contract with a pest control service for regular monthly pesticide applications.

The objectives of this study were to develop dermal-transfer coefficients for young children engaged in their routine activities in their routine environment, to evaluate use of the transfer-coefficient approach for characterizing children’s nonoccupational exposure to pesticides, and to examine children’s activities as a potential determinant of dermal exposure.

## Materials and Methods

### Study subjects.

The child care center selected for monitoring had a long-term contract with a pest control operator to conduct monthly pesticide applications, and detectable levels of esfenvalerate were measured during screening visits (Table 1). In addition, the child care center director was enthusiastic about participating and willing to assist in recruiting individual children.

Two classrooms were selected: an infant classroom for children 6–12 months of age, and a preschool classroom for children 2–3 years of age. We developed background materials, including a recruitment brochure and poster that was displayed at the child care center. Nine children (four or five from each classroom) were recruited for each monitoring session. In this study, we complied with all applicable requirements under U.S. regulations on use of human subjects. Institutional review board approvals were obtained before conducting the monitoring phase of the study. Because no child was intentionally exposed to pesticides for the purposes of this study, risks to the participant were minimal (related only to the burden of wearing the cotton body suit and having hand-wipe samples collected). Participating child care workers, the child care center, and parents/guardians of the participating children all provided written informed consent. The parents of participating children were given $10 for each day of monitoring, and the child care center was given $90 for each day. There were three post–pesticide application monitoring visits.

### Surface sampling.

The child care center director knew in advance that the scheduled pesticide application would be the third Tuesday of each month and called the sampling team on the day of application to confirm that the application would take place. The field team arrived at the child care center in the morning immediately after the application to collect surface samples. Pesticide residues were measured on the floor surfaces from each of the classrooms in areas where children were expected to spend the most time.

During each visit to the child care center, three surface-wipe samples were collected to determine the loading (nanograms per square centimeter) of pesticide residue on hard surfaces in each classroom. Sof-Wick cotton sponges (Johnson & Johnson, New Brunswick, NJ) were saturated with 10 mL isopropanol and wiped across a 1-ft^2^ surface area (929 cm^2^). The wipe samples were stored in a sealed glass jar for subsequent Soxhlet extraction with hexane solvent and analysis by gas chromatography/mass spectrometry (GC/MS).

In addition to the wipe samples, polyurethane foam (PUF) roller samples and surface press samples were also collected on each of the three monitoring visits. Two PUF roller samples were collected using an American Society for Testing and Materials standard method (ASTM 2001). The PUF samples were also stored in a sealed glass jar for subsequent Soxhlet extraction with hexane solvent and analysis by GC/MS.

Surface press samples were collected in 10 locations across the classroom floor using a modified EL press sampler (Edwards and Lioy 1999; U.S. EPA 2001b). A solid base of the same weight as the EL sampler was used with two C_18_-impregnated Teflon extraction disks (3M Empore disks; 3M Corp., St. Paul, MN) as the sampling medium. The surface of a classroom was divided according to surface type: hard (vinyl floor) and soft (carpet or padded areas). We selected five locations on each surface type for sampling. These locations were recorded on a map of the floor area. At each of the 10 locations an individual surface press sample was collected and analyzed separately. In addition, a composite sample was collected for each floor surface type by using one press sampler to collect five consecutive samples at the marked locations without changing the C_18_ disks. Finally, a composite sample for the entire classroom was collected by conducting 10 consecutive presses at each of the marked locations without changing the C_18_ disks. In theory the resulting data would form the basis for constructing a protocol for obtaining a spatial average of the transferable residue across the classroom. In each case, the sampler was applied to the collection surface for 2 min, and the net sampling area of the two disks was 114 cm^2^. After sample collection, the loaded filters were removed and placed in a storage jar. The sample filters were extracted by shaking in 100 mL of the extraction solvent, which was concentrated for analysis by GC/MS.

### Exposure monitoring.

Three postapplication monitoring visits were conducted over the course of 3 consecutive months beginning in July. An attempt was made to monitor each subject for both monitoring sessions in a given visit and to retain each subject for monitoring during subsequent visits. Initially, some of the recruited children were hesitant to participate, so these children were not included in the early monitoring sessions. In addition, some children graduated to older classrooms between visits and were not available for monitoring on subsequent visits. These children were replaced with new recruits from the appropriate classroom.

Monitoring was conducted the day after a reported pesticide application. Recruited children were monitored once in the morning and then again in the afternoon during regular activities for each of the two participating classrooms. During each monitoring session, the children were clothed in full-body 100% cotton body suits (Dharma Trading Company, San Rafael, CA) that had been precleaned by Soxhlet extraction in hexane. The children wore the body suits for 30–60 min during each session. The beginning and ending times were recorded in the field log. At the end of the session, a hand-wipe sample was collected from each child using a gauze pad wetted with alcohol. The hand-wipe samples were stored in jars, and then the suits were removed. Each suit was immediately cut into sections (arms, legs, upper torso, and lower torso), and each section was wrapped in aluminum foil for transfer to the laboratory. The bodysuit sections were sonicated in 500 mL extraction solvent; the solvent was then concentrated for analysis by GC/MS.

**Figure 1:**
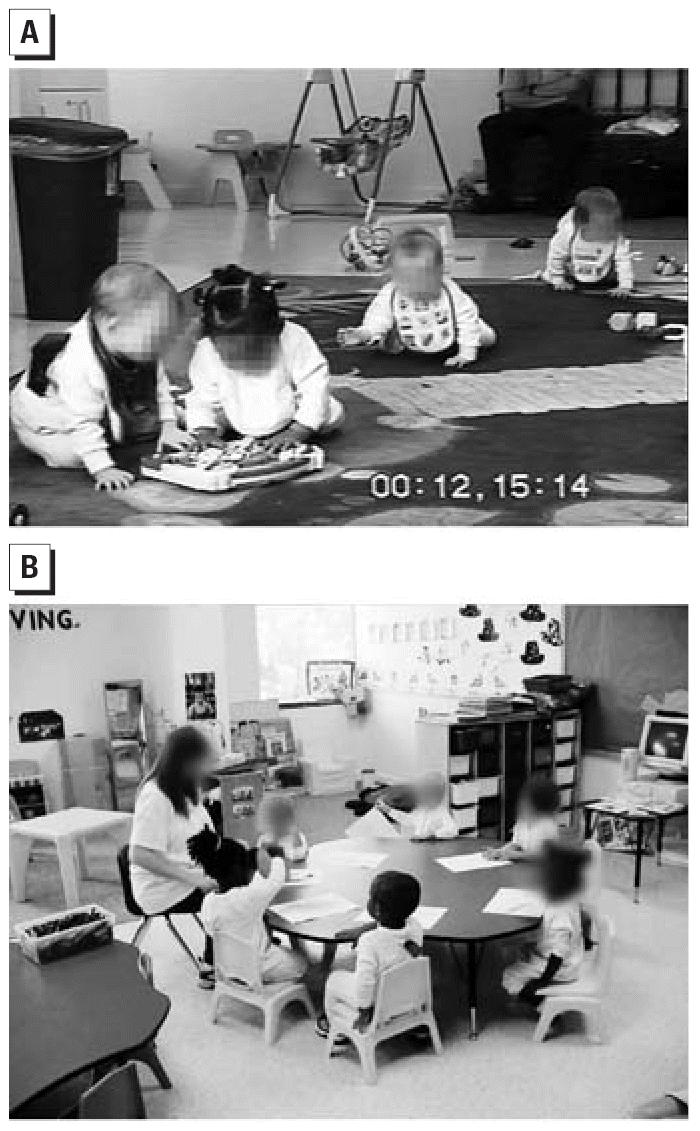
Monitoring session: (*A*) infants and (*B*) preschoolers. Faces have been blurred to protect identities.

A video camera was set up in the corner of each classroom, and a photographer videotaped the children for the duration of each monitoring session. Colored ribbons were fastened to the back of each body suit to facilitate identification of the monitored children in the resulting videotapes. Figure 1 shows a snapshot of a monitoring session in each of the classroom.

### Quality assurance.

Monitoring followed protocols developed specifically for this study in accordance with the study design. An independent auditor audited compliance with these protocols. Accuracy of all analyses was expressed as the percent recovery of the target pesticide from spiked cotton suit sections. All analytical results were measured to be within 30% of the target value. Precision of the bodysuit measurements was a measure of percent relative standard deviation between duplicate samples of the sample aliquot. This measure reflects only the overall variability of the analysis process. All duplicate measurements were within 15%. Of the 220 possible body parts (four sections times 55 body suits), only four were lost because of “soiling” by one of the participants, which results in a measure of 98% completeness. The minimum detectable level (MDL) for the bodysuit measurement was approximately 0.01 ng/cm^2^. Data validation of the final data set consisted of verifying that all required data collections were accounted for, checking for data transcription errors, verification of the data computations, and visual inspection for possible outliers.

### Data analysis.

#### Calculating transfer coefficients.

We used the dermal exposure and surface loading measurements to calculate dermal-transfer coefficients for each monitoring event. The following algorithm was used to calculate the dermal-transfer coefficient (square centimeters per hour) (Fenske 1993):


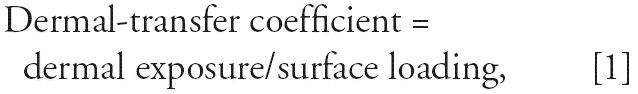


where dermal exposure is the mass of pesticide on the whole-body garment divided by the monitoring duration (nanograms per hour), and surface loading (nanograms per square centimeter) refers to surface-wipe measurements.

For each monitoring visit, we used the exposure measurements (garment loading and associated monitoring duration) collected for all of the children to compute an average dermal exposure. In addition, we used exposure measurements collected in the individual classrooms to calculate a separate average dermal exposure for children in the infant classroom and for those in the preschool classroom. Surface loading data from both classrooms was combined for each visit. We then calculated transfer coefficients using the median value of the surface loading as well as the lowest and highest surface measurements.

#### Evaluating videotapes.

Each taped session was reviewed for the following information:

- Classroom activities: Were children engaged in unstructured free activities or structured group activities?
- Locations of children: Did the children spend time on the floor (carpet/vinyl), in a chair, or standing?
- Activity level of child: Was a child highly interactive with surfaces and other children? Note that this was judged relative to the group for the session.
- Areas of the body suit where contact occurred.
- Unusual factors: Did the child wash or wipe his or her hands? Did he or she wipe hands on the body suit? Was a child playing along walls where surfaces were sprayed?

We considered the following criteria for evaluating activity level from the videotapes for this study. Only a qualitative assessment of these criteria was conducted.

- Range of activity: Does the child spend the entire time in one corner of the room or is the child all over the room?
- Number of activities: Does a child play with one toy or move from one toy to the next?
- Time on the floor or other surface: During circle time, does the child sit the entire time, or squirm on his or her stomach and back? During a table based activity, does the child sit in his or her chair or squirm over the table top?

Based on this assessment, children were classified by activity level. The most obviously active children were classified as high, the least active as low, and the remaining as middle.

### Statistical analysis.

We calculated descriptive statistics for measured loading to the bodysuit sections (arms, legs, upper torso, lower torso), including mean and standard deviation. We used the nonparametric Wilcoxon rank-sum test to investigate the effect of relative activity level on bodysuit loadings, and the Spearman correlation coefficient to investigate agreement between pesticide loading on body suits and hand-wipe concentrations.

We then performed multiple linear regression analysis using the MIXED procedure of SAS (Littell et al. 1996) to identify important determinants of the measured bodysuit loadings. Mixed-effects models were necessary to accommodate the between- and within-person variation in the repeated measurements. A compound symmetry covariance structure with child as the subject-effect was assumed. The four sections of the body suits were included in the model as four levels of a covariate.

We constructed statistical models empirically from the available pool of measured covariates (bodysuit section, visit number, morning or afternoon session, assigned room, age, sampling duration, and relative activity level gleaned from videotapes). Only those covariates with *p*-values < 0.05 were retained. We used Schwarz’s Bayesian information criterion (Littell et al. 1996) to select the model that best fit the data.

We evaluated between- and within-person variability using logged variance components α ^2^_B_ and α^2^_W_ estimated from the null model (random effects only), performed separately for each dosimeter section. We used the intra-class correlation coefficient (ICC)—the ratio of the between-person component to the total variance—to infer the proportion of total variability that is due to differences among individuals rather than differences in the environment from one sampling occasion to the next. Between- and within-person geometric standard deviations (GSDs) were estimated from the logged variance components.

## Results

### Surface sampling.

Table 1 presents the results of the surface loadings measured at the child care center. The surface loadings varied by a factor of 10–40 for measurements at the same location across the three monitoring visits in the preschool room and by only up to a factor of 3 for the infant room. These limited sampling results do not demonstrate any clear pattern from visit to visit (e.g., a decrease in surface concentrations with each visit) nor that either room had more pesticide residue on the floor than the other.

### Exposure monitoring.

**Figure 2:**
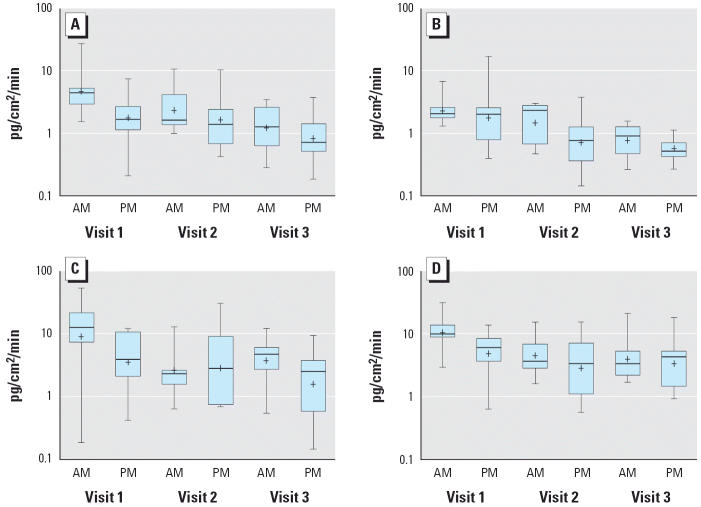
Box plots of bodysuit section loadings per unit time: (*A*) arms, (*B*) upper torso, (*C*) legs, and (*D*) lower torso. Abbreviations: AM, morning; PM, afternoon.

Detectable levels of esfenvalerate were measured on at least three sections of all of the garments worn by children in each of the monitoring sessions. Summary results of dermal exposure monitoring are presented in Table 2 and Figure 2. Table 2 illustrates that, on average, the morning sessions recorded higher loadings (picograms per square centimeter) and loadings decreased with each visit. Figure 2 presents the data by body part (arm, upper torso, legs, and lower torso). Figure 2 illustrates the very clear difference between the morning and afternoon loadings, with the afternoon sessions being much lower than the morning sessions in each visit for each section of the body suit. It can also be seen that measured loadings were generally highest for the first visit and lowest for the third. Although these results demonstrate that pesticide residues transfer from surfaces to the body suits after a professional application, the amount that is available for dermal transfer is unknown.

**Figure 3:**
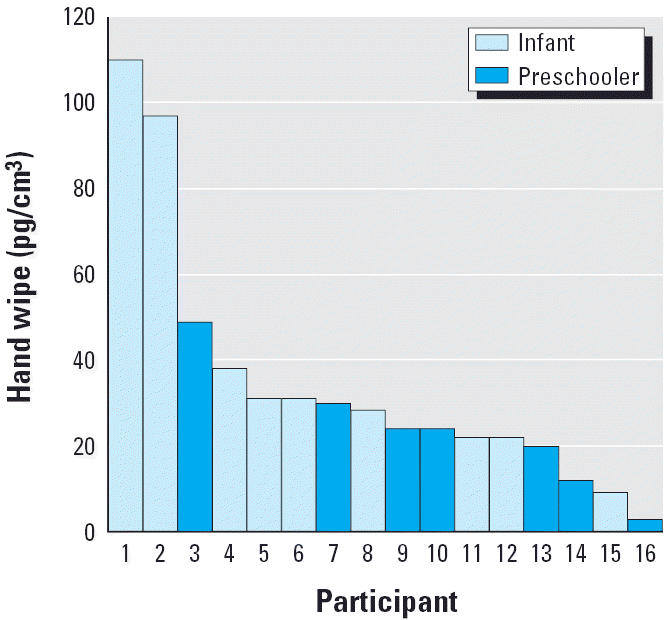
Hand-wipe loadings above MDL among infants and preschoolers, sorted in descending order, illustrating that the highest loadings were typically from infants and the lowest typically from preschoolers.

Of the 50 hand-wipe samples that were collected and analyzed, 34 (68%) were below the MDL. The infants had 1.5 times as many values (36%) above the MDL as did the preschool children (24%). This is consistent with the higher loadings measured on the infant body suits. Of the 16 measurable hand-wipe values, six of the highest eight values were measured from the infants (Figure 3). In the session with the highest proportion of detectable measurements for the preschool children, the main activity consisted of sitting on the carpeted floor while being read to by the teacher. This session was also the longest, about 15–20 min longer than the others.

### Data analysis.

#### Transfer coefficients.

Calculated dermal-transfer coefficients are presented in Table 3. Overall, the dermal-transfer coefficients range from 7.5 to 6,200 cm^2^/hr. In general, transfer coefficients for infants were slightly higher than those for the preschool-age children. Results of the transfer coefficients for visit three were significantly affected by one very high surface-wipe sample collected during that visit.

#### Videotapes.

Based on qualitative review of the videotape data, activity level appears to be a strong indicator of bodysuit loading based on relative comparison with activity level of other children in a group. Comparison of overall group activity level from one session to the next showed that children were generally more active in the morning sessions. This observation was consistent with the higher loadings of pesticide measured on the body suits collected from the morning monitoring sessions. Specific observations for infants and preschoolers are discussed separately.

During all of the monitoring sessions, the infants spent the time engaged in unstructured activity on the classroom floor. Most children stayed on the play mat (carpet) in the center of the room. More mobile children moved off the mat (crawled or squirmed on stomach) onto the surrounding vinyl floor. The most developmentally advanced child, monitored only in session 1 before being moved to the next level classroom, crawled out of view of the camera and spent most of the morning monitoring period crawling, pulling up, and walking along the edge of the classroom walls. Consistent with measured bodysuit loadings, most contact for the infants was on the lower torso and legs, with more limited contact on the lower arms when crawling. The infants were generally less active in the afternoon monitoring sessions, which took place as the children awoke from their naps. This, too, is consistent with measured exposure levels. Bodysuit loadings for the most active and least active infants is presented in Table 4.

For the preschool children, it is more difficult to generalize about each child’s activity patterns from the videotape data because there were many times when the child was out of view of the camera, especially during unstructured times. However, in general, the most active children did in fact have the highest loadings (Table 5). General classroom activity varied during each visit. The greatest level of activity was observed during visit 1, and all activity was unstructured (free play). In visit 2, about half of the morning session was free play, followed by time sitting at tables for reading and art. In the afternoon, all activities took place at the table. In visit 3, the morning began with free play, followed by story time on the floor; the session ended at the table for reading and art. In the afternoon, all time was unstructured, but little floor contact was observed. Thus, general activity level in visit 2 was lower than for visit 1, and the general activity level during visit 3 was the lowest of the three.

### Statistical analysis.

Multiple regression analysis produced several competing models. The model that best predicted bodysuit loading (picograms per square centimeter per second) is listed in Table 6. This model includes bodysuit section, visit, session (morning or afternoon), and relative activity level. The magnitudes of the effect estimates for section, visit, and session correspond well with the summary statistics presented in Table 2. Surface loadings measured on the floors were not a significant predictor of garment levels, casting some doubt on the adequacy of the surface loading measurements. The statistical significance of activity, even in a model that controls for classroom, suggests that activity level within age groups may be as important as age-related differences in the type of activities performed.

The between- and within-person variance components for loadings on each of the four garment sampler sections are listed in Table 7 along with the ICCs and the GSDs. The within-person component is large for all four sections, and the low ICCs suggest that the loading is more dependent on factors related to changing conditions in the environment than on traits specific to the individuals being monitored. The loadings on the legs and the lower torso display the most consistency from one sampling occasion to the next based the ICCs of 0.40 and 0.39, respectively. The loadings on the legs have the highest total variability, as well as the highest average values.

In the one session for the preschool children when all children had detectable hand-wipe levels for comparison, the relative ordering of the total body loading and the hand-wipe levels shows good agreement. There were two sessions for the infants when at least half of the measurements were detectable. The relative order of the hand-wipe concentrations during these sessions again shows good agreement with the relative order of the bodysuit loadings. The overall Spearman rank correlation is rho = 0.84 (*p* = 0.0007).

## Discussion

Results of this study provide a set of transfer coefficients that can be used to characterize dermal exposure of young children to pesticides in a nonoccupational setting. The values for dermal-transfer coefficients developed in this study were compared with the default values currently used by the U.S. EPA’s OPP to assess residential exposure to pesticides. Although there are difficulties associated with comparing transfer coefficients developed using different surface sampling techniques, results of this work suggest that the default assumption used by the U.S. EPA OPP is reasonable. We found the upper-range values resulting from this study (5,900–6,200 cm^2^/hr) to be similar to the default value used by the U.S. EPA to assess children’s dermal exposures resulting from contact with indoor surfaces (6,000 cm^2^/hr). Application of this type of transfer coefficient assumes that loading of pesticide on the skin surface will increase in a linear fashion with time. This is not likely to be the case. However, we were not able to evaluate the significance of the exposure time frame in this study because of the small differences in the monitoring times.

Results of this study also demonstrate the potential for children to be exposed to pesticides through the dermal route after use of pesticides in a child care environment. All of the body suits collected contained measurable levels of esfenvalerate. However, this study does not characterize the potential for the pesticides to be absorbed at levels that might be of concern for health effects. Surface-to-skin transfer of contaminants and subsequent absorption over the time course of an exposure is a complex process. Once on the skin, pesticide residues and contaminated particles can be transferred back to the contaminated surface during subsequent contact, lost by dislodgment or washing, or transferred into the body by percutaneous absorption or hand-to-mouth activity. These mechanisms of transfer and the associated rates will be different for the cotton body suits than for skin. Additional research is required to characterize the relationship between loading of pesticide on a cotton whole-body garment sampler and amount of pesticide that can be absorbed before this approach can be used to directly estimate risk (Souter et al. 2000).

Additional challenges associated with interpreting results of this type of dermal exposure study include the significant inter-and intraindividual variation in measured bodysuit loadings, the impact of surface measurements on development and use of dermal-transfer coefficients, and importance of classifying activity to predict exposure. As mentioned above, the dermal-transfer coefficient approach for assessing dermal exposure was developed for use in agricultural settings. A recent review of temporal variability in dermal exposure (Kromhout and Vermeulen 2001) investigated between- and within-person variability of occupational dermal exposure measurements, including measurements of surface-to-skin transfers using dermal pads. The authors reported median between- and within-person GSDs of 1.5 and 2.0, respectively. The between- and within-person GSDs in the child care setting are similar (1.4–2.0 and 2.2–2.8, depending on the body part) to those in the agricultural/industrial setting.

A major difficulty with interpreting and using the transfer coefficients developed in this study is associated with the poor results obtained from surface sampling. As noted above, results of the transfer coefficients for visit 3 were significantly affected by one very high surface-wipe sample collected during that visit. This result has an important impact on the ability to use transfer coefficients developed during session 3 to predict exposures from the earlier visits.

It is critical to understand that application of a transfer coefficient developed in one study to estimate exposure in a second study requires that the surface sampling method used in each study be the same. Methods for sampling surface residues available for dermal transfer have been under development for years. Even so, existing measures are susceptible to high variability (Fenske 1993). Some of this variability can be attributed to the nonhomogeneity of pesticide residues in the residential environment. But there is still significant variation due wholly to limitations of the sampling methodologies. In this study, we had hoped to develop and evaluate a protocol for collecting an aggregate surface sample representative of the residue available for transfer to a child. Because of failure of the sampling method (surface press sampler) to collect detectable levels of esfenvalerate, we were not able to accomplish this.

If the transfer-coefficient approach for characterizing dermal exposure is to be truly effective for assessing risks in a residential environment, further research is required to develop and verify standard methods and protocols for measuring compounds on surfaces that are representative of nonuniform distributions found in residential environments, representative of residues and particles available for transfer to skin, and able to measure low levels found in environmental situations. Currently, the U.S. EPA’s Office of Research and Development suggests that multiple individual samples (at least three) should be collected from various areas in the micro-environment where the child is in contact with surfaces and that the locations selected should be representative of where the child spends his or her time (U.S. EPA 2001b).

Finally, because human behavior is variable and difficult to quantify, consideration should be given to developing a better qualitative understanding of the behavioral determinants of exposure. Results of this study suggest that the most active children also tend to be the children with the highest potential dermal exposure. However, we currently do not have a validated approach for identifying and classifying children based on activities and behavior that could be used to inform exposure assessment. Research is required to identify and apply tools developed in the social science arena by specialists in child development and environmental anthropology to characterize and classify behaviors that are major determinants of exposure. Adapting assessment tools developed to characterize a child’s temperament may be one possible approach. Carey (2003) discusses nine behavioral style traits that constitute a child’s temperament, which include activity level, intensity, persistence, distractibility, approach/withdrawal (initial reaction to new stimuli), and sensory threshold (sensitivity). Field experience and qualitative assessment of videotape data collected in our study suggest that several of these style traits may directly influence a child’s potential for dermal exposure. It is possible that addition of several targeted questions for a parent before field monitoring may improve our ability to characterize and classify children based on their potential to be exposed to environmental contaminants.

## Figures and Tables

**Table 1:** Surface loadings of esfenvalerate measured at child care center using alcohol wipes (ng/cm^2^).

	Preschool room			Infant room		
Date	Near exterior door	Near restrooms	Center of room	Near interior door	Changing table	Notes
10/18/00	16	1.3	0.72	—	—	Application 10/17/00 screening visit
6/15/01 (preapplication)	20	1.8	—	—	—	Application 6/19/01 screening visit
6/20/01	1.1	1.4	0.71			
6/21/01	1.4	3.8	0.48			
6/25/01	7.6	42	0.64			
7/18/01	3.2	16	—	0.48	51	Application 7/17/01 monitoring visit
8/22/01	6.6	2.7	—	0.47	15	Application 8/21/01 monitoring visit
9/19/01	120	22	—	0.56	27	Application 9/18/01 monitoring visit

—, no samples collected.

**Table 2:** Whole-body garment sampler loadings [average (SD)].

	Infants		Preschool children	
Visit	pg/cm^2^	pg/cm^2^/min	pg/cm^2^	pg/cm^2^/min
7/18/01				
Morning	290 (218)	9.4 (6.1)	311 (310)	7.7 (7.1)
Afternoon	123 (56.9)	4.1 (1.9)	174 (130)	4.4 (3.2)
8/22/01				
Morning	196 (160)	4.9 (4.0)	134 (52.1)	2.3 (1.0)
Afternoon	119 (99.6)	4.8 (4.0)	87.5 (105)	2.3 (2.6)
9/19/01				
Morning	151 (102)	4.0 (2.9)	105 (55.5)	1.8 (1.0)
Afternoon	84.8 (26.5)	2.6 (0.9)	82.1 (74.9)	1.8 (1.6)

**Table 3:** Dermal-transfer coefficients [median (range)].

Visit	Average dermal exposure (ng/hr)	Surface loading (ng/cm^2^)	Transfer coefficient (cm^2^/hr)	Transfer coefficient^a^ (cm^2^/hr)
Visit 1				
Infants	1,800	1.8 (0.48–51)	1,000 (35–3,800)	1,700 (59–6,200)
Preschoolers	1,710		950 (34–3,600)	1,600 (56–5,900)
All children	1,760		980 (34–3,700)	1,600 (58–6,100)
Visit 2				
Infants	1,180	1.6 (0.47–15)	740 (79–2,500)	1,200 (130–4,200)
Preschoolers	700		440 (47–1,500)	730 (78–2,500)
All children	902		560 (60–1,900)	940 (100–3,200)
Visit 3				
Infants	937	11 (0.56–120)	85 (7.8–1,700)	140 (13–2,800)
Preschoolers	539		49 (4.5–960)	82 (7.5–1,600)
All children	726		66 (6.0–1,300)	110 (10–2,200)

a Adjusted for potential transfer to hands and feet based on the study by Ross et al. (1990), in which approximately 40% of residue was transferred to garment on hands and feet.

**Table 4:** Comparison of bodysuit loadings and activity level for infants.

	Most active		Least active	
Visit	Subject ID	pg/cm^2^/min	Subject ID	pg/cm^2^/min
Visit 1				
Morning	CI, EI	20.0, 7.6	DI	4.0
Afternoon	CI	4.7	BI	2.7
Visit 2				
Morning	AI, GI	9.4, 7.0	FI, HI	1.5, 1.6
Afternoon	AI	9.5	FI	0.6
Visit 3				
Morning	GI	8.2	AI	1.7
Afternoon	GI	3.7	FI	0.6
Average		8.8		1.8

ID, identifier.

**Table 5:** Comparison of bodysuit loadings and activity level for preschool children.

	Most active		Least active	
Visit	Subject ID	pg/cm^2^/min	Subject ID	pg/cm^2^/min
Visit 1				
Morning	AP	15.7	CP	1.9
Afternoon	DP	7.9	CP, EP	1.5, 1.9
Visit 2				
Morning	DP	3.8	AP, BP	1.5, 1.6
Afternoon	DP	7.5	AP, FP	0.5, 0.8
Visit 3				
Morning	DP	3.0	FP	0.6
Afternoon	DP, FP	3.9, 3.0	AP, GP	0.6
Average		6.4		1.2

ID, identifier.

**Table 6:** Results of multiple regression modeling of measured bodysuit pesticide loading [log(pg/cm^2^/sec)].

Effect	Level	Estimate	*p*-Value
Intercept		−1.43	< 0.0001
Bodysuit section	Arms	0.46	< 0.0001
	Legs	1.05	
	Lower	1.35	
	Upper	0	
Visit	1	0.87	0.0006
	2	0.31	
	3	0	
Session	Morning	0.44	0.0006
	Afternoon	0	
Activity level	High	1.36	< 0.0001
	Middle	0.65	
	Low	0	
Classroom	Infant	0.38	0.0386
	Preschool	0	

**Table 7:** Measures of between- and within-person variability for loading on individual bodysuit sections.

	Arms	Upper	Legs	Lower
Logged between-person variance	0.26	0.04	0.67	0.37
Logged within-person variance	0.76	0.76	1.02	0.59
ICC	0.25	0.05	0.40	0.39
GSD, between	1.7	1.2	2.3	1.8
GSD, within	2.4	2.4	2.7	2.2

## References

[b1-ehp0114-000264] ASTM 2001. Standard D 6333-98. Standard practice for collection of dislodgeable residues from floors. In: Annual Book of ASTM Standards. Vol 11.03. West Conshohoken, PA:American Society for Testing and Materials.

[b2-ehp0114-000264] Black KG, Fenske RA (1996). Dislodgeability of chlorpyrifos and fluorescent tracer residues on turf: comparison of wipe and foliar wash sampling techniques. Arch Environ Contam Toxicol.

[b3-ehp0114-000264] Carey WB (2003). Children’s temperaments influence the impact of environmental risks. J Children’s Health.

[b4-ehp0114-000264] Cohen Hubal EA, Sheldon LS, Burke JM, McCurdy TR, Berry MR, Rigas ML (2000a). Exposure assessment for children: a review of the factors influencing exposure of children, and the data available to characterize and assess that exposure. Environ Health Perspect.

[b5-ehp0114-000264] Cohen Hubal EA, Sheldon LS, Zufall MJ, Burke JM, Thomas K (2000b). The challenge of assessing children’s residential exposure to pesticides. J Expo Anal Environ Epidemiol.

[b6-ehp0114-000264] Edwards RD, Lioy PJ (1999). The EL sampler: a press sampler for the quantitative estimation of dermal exposure to pesticides in housedust. J Expo Anal Environ Epidemiol.

[b7-ehp0114-000264] Fenske RA (1993). Dermal exposure assessment techniques. Ann Occup Hyg.

[b8-ehp0114-000264] Food Quality Protection Act of 1996 1996. Public Law 104–170.

[b9-ehp0114-000264] Formoli TA 1996. Estimation of Exposure of Persons in California to Pesticide Products that Contain Propetamphos. HS-1731. Sacramento:California Environmental Protection Agency.

[b10-ehp0114-000264] Kromhout H, Vermeulen R (2001). Temporal, personal and spatial variability in dermal exposure. Ann Occup Hyg.

[b11-ehp0114-000264] Littell RC Milliken GA Stroup WW Wolfinger RD 1996. SAS System for Mixed Models. Cary, NC:SAS Institute Inc.

[b12-ehp0114-000264] Ross J, Fong HR, Thonsinthusak T, Margetich S, Krieger R (1991). Measuring potential dermal transfer of surface pesticide residue generated from indoor fogger use: using the CDFA roller method. Chemosphere.

[b13-ehp0114-000264] Ross J, Thongsinthusak T, Fong HR, Margetich S, Krieger R (1990). Measuring potential dermal transfer of surface pesticide residue generated from indoor fogger use. Chemosphere.

[b14-ehp0114-000264] Souter A, Semple S, Aitken RJ, Robertson A (2000). Use of patches and whole body sampling for the assessment of dermal exposure. Ann Occup Hyg.

[b15-ehp0114-000264] U.S. EPA 1997. Standard Operating Procedures (SOPs) For Residential Exposure Assessment. Washington, DC:Office of Pesticide Programs, U.S. Environmental Protection Agency. Available: http://www.epa.gov/oppfead1/trac/science/trac6a05.pdf [accessed 6 January 2006].

[b16-ehp0114-000264] U.S. EPA 1999. Overview of Issues Related to the Standard Operating Procedures (SOPs) for Residential Exposure Assessments. Washington, DC:Office of Pesticide Programs, U.S. Environmental Protection Agency. Available: http://www.epa.gov/scipoly/sap/1999/september/resid.pdf [accessed 13 September 2005].

[b17-ehp0114-000264] U.S. EPA 2001a. Policy Number 12, Regarding: Recommended Revisions to the Standard Operating Procedures (SOPs) for Residential Exposure Assessments. Washington, DC:Office of Pesticide Programs, U.S. Environmental Protection Agency.

[b18-ehp0114-000264] U.S. EPA 2001b. Draft Protocol for Measuring Children’s Non-occupational Exposure to Pesticides by All Relevant Pathways. EPA/600/R-03/026. Research Triangle Park, NC:Office of Research and Development, U.S. Environmental Protection Agency.

[b19-ehp0114-000264] Vacarro JR Nolan RJ Murphey PG Berbrich DB 1996. ASTM STP 1287. In: American Society for Testing and Materials (Tichenor BA, ed). West Conshohoken, PA:American Society for Testing and Materials, 166–183.

